# Analytical Investigations of XIX–XX Century Paints: The Study of Two Vehicles from the Museum for Communications of Frankfurt

**DOI:** 10.3390/molecules28052197

**Published:** 2023-02-27

**Authors:** Andrea Macchia, Lisa Maria Schuberthan, Daniela Ferro, Irene Angela Colasanti, Stefania Montorsi, Chiara Biribicchi, Francesca Irene Barbaccia, Mauro Francesco La Russa

**Affiliations:** 1 YOCOCU (Youth in Conservation of Cultural Heritage), Via T. Tasso 108, 00185 Rome, Italy; 2 Museum for Communication Frankfurt, Schaumainkai 53, 60596 Frankfurt am Main, Germany; 3 Department of Biology, Ecology and Earth Sciences DIBEST, University of Calabria, Via Pietro Bucci, Arcavacata, 87036 Rende, Italy

**Keywords:** protective coating, paint, binder, pigment, characterization, non-invasive techniques, FT-IR ATR, SEM-EDS

## Abstract

Over the centuries, humans have developed different systems to protect surfaces from the influence of environmental factors. Protective paints are the most used ones. They have undergone considerable development over the years, especially at the turn of the 19th and 20th centuries. Indeed, between the two centuries, new binders and pigments have been introduced in the constituent materials of paints. The years in which these compounds have been introduced and spread in the paint market allow them to be defined as markers for the dating of paints and painted artifacts. The present work is focused on the study of the paints of two vehicles of the Frankfurt Museum of Communication, i.e., a carriage and a cart, that was designed for the German Postal and Telecommunications Service roughly between 1880 and 1920. The characterization of the paints was performed through in situ non-invasive techniques, i.e., portable optical microscopy and multispectral imaging, and laboratory non-destructive techniques, i.e., FT-IR ATR spectroscopy and SEM-EDS. The analytical investigation and the comparison with the data reported in the literature allowed us to determine the historicity of the paints, which are all dated before the 1950s.

## 1. Introduction

Over the centuries, several protective paints that could be applied to artifacts have been produced.

As to vehicles used for transportation, the function of the pictorial coating was protection from the outdoor environment and their consequent degradation [1,2,3,4]. Currently, protective paints are highly specific according to the substrate on which they have to be applied while, until the first half of the 20th century, the choice was based on the type of environment in which they would have been placed: namely indoor or outdoor environments [5,6,7].

Protective paints consist of three components: binders, dyes or pigments, and additives. Over the centuries, especially at the turn of the XIX and XX centuries, several developments in the production of paints were mainly linked to the introduction of new binders and pigments [4,6,7,8,9,10,11]. These developments are attributable to two main factors that have characterized the centuries: the Industrial Revolution and the automobile’s mass production. These two events led to the growth of the first paint factories in the early 19th century, which started developing linseed oil paints, galvanizing processes, and TiO_2_-based pigments in 1918. Linseed oil is a natural oil that is characterized by a concentration of linoleic acid-fatty acid whit formula C_18_H_32_O_2_-around 70%, and consequently has a high number of double bonds. This characteristic makes linseed oil have a high drying power, which is one of the main reasons for its wide use in paintings and the production of paints until the first half of the 20th century [12,13]. With the introduction of synthetic resins, advancements in the coating industry quickly followed to meet market demand. Thus, in the middle of the XX century, natural oils that were traditionally used in paint formulations were being replaced by synthetic resins. Therefore, the identification of an oily medium in a coating may suggest that it was produced before 1950, while the presence of synthetic resins suggested a later dating. Furthermore, alkyd paints almost completely replaced oil paints in the second half of the XX century [4,6,7,11,13,14,15].

Pigments can be used as markers as well. Among them, the most important ones that have this role are lead-based pigments, zinc white, lithopone, and titanium white. Lead-based pigments, especially lead white (basic lead carbonate 2PbCO_3_·Pb(OH)_2_), has been traditionally used to produce paint matrices. Due to the discovery of their toxicity, their use decreased between the XIX and XX centuries [4,6,7,16,17,18]. In the years leading up to World War II, consumers paid more attention to health and environmental risks, and paint manufacturers began to study safer alternatives to replace lead and other toxic elements.

Zinc white (ZnO) was discovered in the late 1700s; lithopone, a mixture of 70–72 wt% of barium sulfate (BaSO_4_) and 30–28 wt% of zinc sulfide (ZnS), was discovered in the late 1800s. They were commonly used to replace lead white in the first half of the XX century [4,6,7,9,14,15,16,18]. In 1918, the first titanium white was produced as a mixture of 30% titanium dioxide (TiO_2_) and barium sulphate (BaSO_4_). Soon, titanium white started to be the most widely used white pigment in paint matrices of titanium white (TiO_2_). Indeed, a study carried out by the International Agency for Research on Cancer (IARC) highlights an increase in the paints produced with titanium white (about 80%) and a decrease in paints produced with lead white or lithopone in these years [18]. Consequently, the presence of this pigment allows the paint to be dated after that year and with greater probability after 1940 when the use of white titanium spread. 

Other markers can be defined through the evolution of colored pigments and dyes [6,7,16]. Until the first half of the 1900s, multiple pigments were used for the formulation of paints. In the XIX century, new pigments were introduced to the market, such as synthetic ultramarine blue (Na_8–10_Al_6_Si_6_O_24_S_2–4_), zinc and lead chromates (ZnCrO_4_ or PbCrO_4_), cadmium sulfides (pigments containing CdS), and Paris green (Cu(C_2_H_3_O_2_)_2_·3Cu(AsO_2_)_2_). Their presence suggests that the paints were produced before the 1950s. The use of these pigments decreased progressively due to the discovery of the toxicity of some of them, but especially due to the spread of synthetic dyes. Synthetic compounds such as aniline (an aromatic compound, C_6_H_7_N), azo dyes (organic compounds characterized by the functional group R-N=N-R′, where R and R′ are often aryl groups), and phthalocyanines (macrocyclic organic compounds whose formula is (C_8_H_4_N_2_)_4_H_2_), became the most widely used compounds for paints. Therefore, their presence allows the paints to be dated after the 1950s [4,6,7,16].

The coatings’ composition allows for the acquisition of useful information for the dating of objects and artifacts. However, paints are composed of different constituents, such as binders, pigments, and other fillers. Additionally, several chemical compounds have been used for their production over the centuries. Thus, their characterization is not simple and requires a multi-analytical approach [19]. 

A preliminary study is generally required, especially if several layers overlap. Optical microscopy (OM) is commonly used for this analysis and allows for the acquisition of information on the object’s stratigraphy [19,20]. Additionally, multispectral imaging is often used in the cultural heritage sector since it is a non-invasive technique based on ultraviolet, visible, and near-infrared radiation. It allows more details of the surface to be obtained, such as alterations due to aging, and to discriminate any different constituent materials [21,22,23].

As for the characterization of the chemical composition of paints, numerous techniques can be used. Mass spectroscopy techniques, such as laser ablation-inductively coupled plasma-mass spectrometry (LA-ICP-MS), have been useful for obtaining trace-level information. Chromatographic techniques, such as pyrolysis–gas chromatography–mass spectrometry (Py-GC-MS), allow information to be obtained on the polymer fraction, namely the binder. Furthermore, X-ray fluorescence spectrophotometry (XRF) is used for elemental analysis, while X-ray diffractometry (XRD) allows for the determination of the inorganic fractions’ composition and structure [19,20,24,25,26,27,28,29,30,31,32,33].

Despite the multiple possibilities, FT-IR spectroscopy, Raman spectroscopy, and SEM-EDS appear to be the most commonly used analytical techniques in the cultural heritage sector [19,20,24,25]. FT-IR and Raman spectroscopies are non-destructive techniques, often not requiring sample preparation. The first allows for the characterization of the organic fraction and only part of the inorganic one. The second allows for the identification of the inorganic component and, in particular, the metal compounds. However, the latter is often affected by the fluorescence phenomenon, which does not allow an interpretable spectrum to be obtained [19,24]. Furthermore, SEM-EDS allows information to be acquired on the morphological aspect of the samples, through secondary electrons, and on the elemental composition of the materials, by exploiting X-rays emitted by the sample [20,24]. 

**Figure 1:**
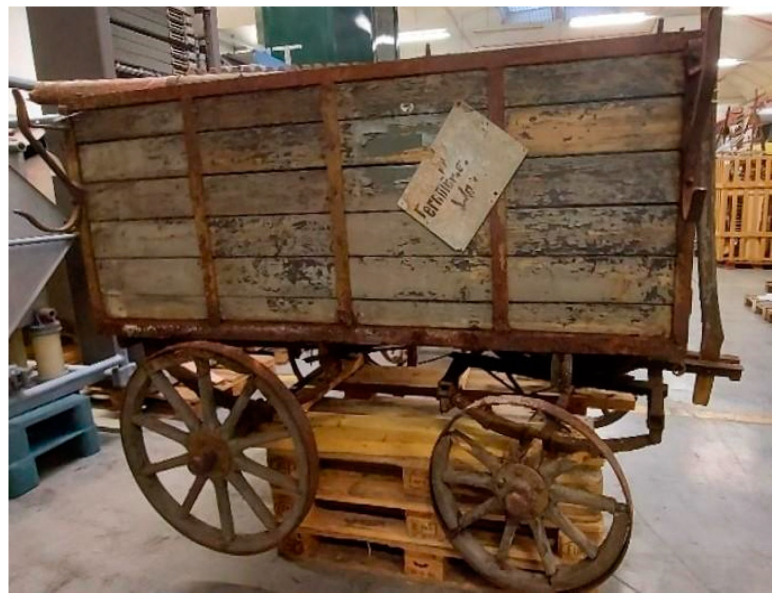
Image of the cart for telecommunication service.

This work is focused on the study of the protective paints of two vehicles belonging to the Museum für Kommunikation in Frankfurt and used by the German postal service at the turn of the two centuries. The first one is a cart for telecommunications services and is dated approximately 1920–1940 (Figure 1). This cart was constructed in the Reichspost era and was continuously used by the postal service also during the German Democratic Republic (GDR). The cart was found in the garden of a former postal employee in 2015. It was partially sunk into the ground, where it presumably stood for decades after having been taken out of service.

**Figure 2:**
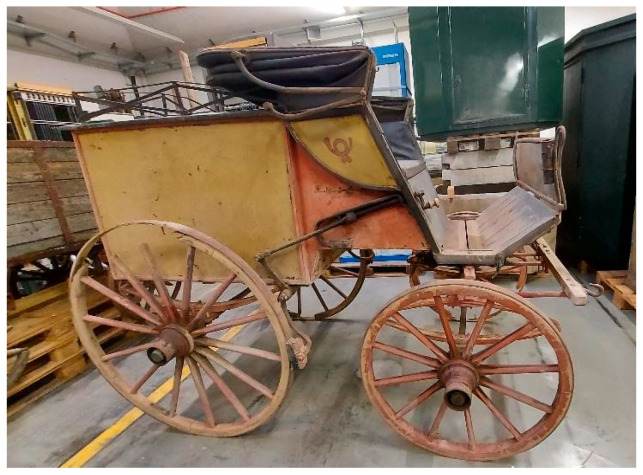
Image of the carriage of the family King.

The second vehicle is a carriage of the German postal service, dated around 1880 (Figure 2). The carriage was found in 1999 when the King’s family house was demolished. It is currently stored at the Museum für Kommunikation in Frankfurt. It has been probably used for the postal service between Schramberg and Rottwell until 1910. 

The main purpose of this work was the characterization of the inorganic and organic fractions of the vehicles’ paints through the use of pigments as markers to allow the discrimination between original and non-original materials. A preliminary study of the surfaces has been carried out through the use of the multispectral imaging system and portable optical microscopy. Both these techniques have enabled the selection of more significant sampling areas to be analyzed with SEM-EDS and FT-IR ATR spectroscopy. Additionally, Raman spectroscopy analyses were performed. However, they were inconclusive due to fluorescence interference and are thus not reported in this paper. This study was thus aimed at identifying and dating the constituent materials by using analytical techniques that are available and easily implementable in most museums’ laboratories for the identification of object materials. For this reason, specific techniques were used to assess the effectiveness of their combined application, defining a replicable operational methodology. This approach can have useful applications in many museums with relatively modern objects that can be characterized, such as vehicles of various natures, with a high historical value. Furthermore, the study aimed at detecting the areas requiring subsequent restoration intervention.

## 2. Results

### 2.1. Multispectral Imaging

#### 2.1.1. The Cart

The multispectral images of the cart’s different areas show the different fluorescence that was induced by three layers of paint (Table 1).

**Figure 3:**
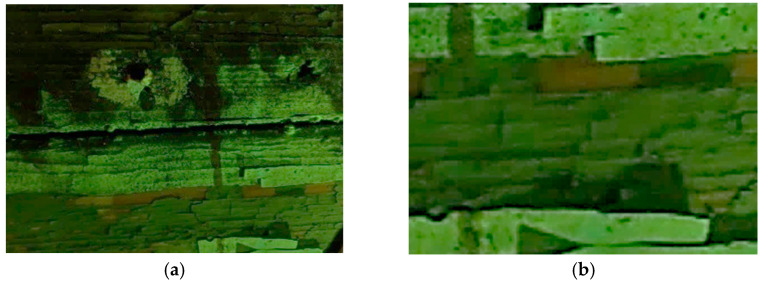
(**a**) Details of the UV fluorescence image of the paints on the right panel; (**b**) Enlargement (10×) of the area.

The outermost layer, which is light grey in VIS light, is characterized by a low yellowish fluorescence that contrasts with the fluorescence induced on the surface of the intermediate grey-greenish layer. The dark grey layer in contact with the wooden support does not show any fluorescence (Figure 3).

**Figure 4:**
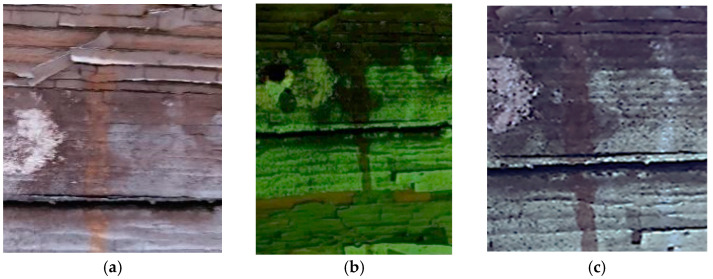
Details of the iron cart structure: leakage due to corrosion; (**a**) Image in visible light; (**b**) Image in UV fluorescence; (**c**) Image in IR reflectography.

Moreover, the investigation allowed the presence of some areas to be highlighted that appeared reddish in both visible and UV light while showing a black color in IR reflectography (Figure 4).

These areas are related to the leakage that resulted from the degradation of the cart’s iron structure, the solubilization of corrosion products, and the pouring of iron–ions-rich solutions.

#### 2.1.2. The Carriage

The analysis performed by multispectral imaging in UV light showed a bluish-yellow ultraviolet UV fluorescence spread over the entire surface of the carriage (Table 2). Such fluorescence could be due to an oil-based paint that aged over time. The UV fluorescence also allowed the widespread presence of cracks in the paint layer to be highlighted, as well as localized corrosion in several areas of the metal panels that appeared darker.

**Figure 5:**
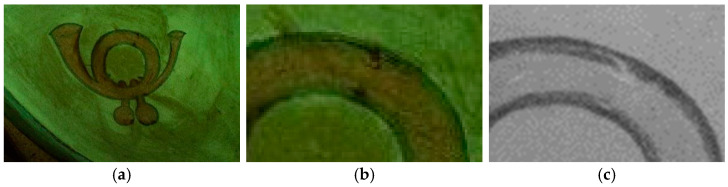
(**a**) Symbol panel of the right side of the carriage in UV fluorescence; (**b**) Enlargement (10×) of the area in UV fluorescence; (**c**) Enlargement (10×) of the area in IR reflectography.

The multispectral investigation performed on the symbol present on the right and left side of the carriage underlined an uneven response under UV radiation (Figure 5a,b). Indeed, darker areas could be noticed, which appeared to be lighter in IR reflectography (Figure 5c). The areas in which the fluorescence is less marked (darker in the photo) present a greater reflection of the IR radiation, and these areas appear lighter. These differences can be presumably related to the loss of the finishing layer.

**Figure 6:**
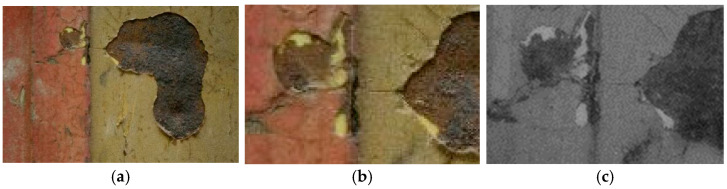
(**a**) Image of the rear panel of the carriage collected under visible light; (**b**) Enlargement (10×) of the area in VIS; (**c**) Enlargement (10×) of the area in IR reflectography. The chromatic differences between the two yellow paints, in visible light and their different reflection of the IR radiation are highlighted.

On the rear panel (Figure 6), the IR reflectography allowed the presence of two yellow paints to be noticed that are characterized by different reflections of the IR radiation.

The more superficial layer appears darker in IR, while the underlying one shows a lighter tone due to the greater reflection of infrared radiation. These characteristics are attributable to the use of different paints. The same feature was also found in the rear axle of the wheels.

### 2.2. Portable Optical Microscopy

#### 2.2.1. The Cart

**Figure 7:**
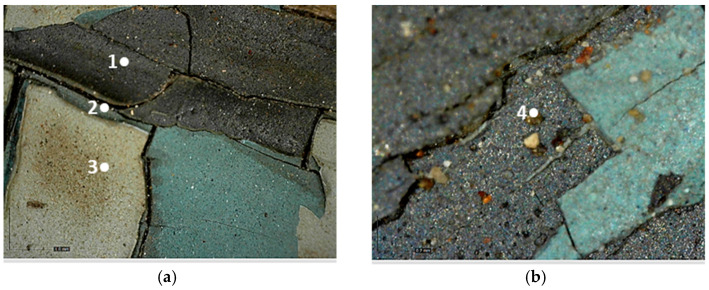
Optical microscope images of the cart’s stratification. (**a**) Image collected at 50× magnification in which the dark grey (1), greenish grey (2), and light grey (3) layers are visible; (**b**) Image collected at 200× magnification where the deposited particulate is present (4).

The investigation was performed using the optical microscope and allowed the stratigraphy of the grey cart to be identified alongside the presence of a deposited particulate on the surface (Figure 7a).

The layers, starting from the innermost one to the outermost one, can be described as follows:

1. Dark grey layer.
2. Greenish grey layer.
3. Light grey layer.

#### 2.2.2. The Carriage

**Figure 8:**
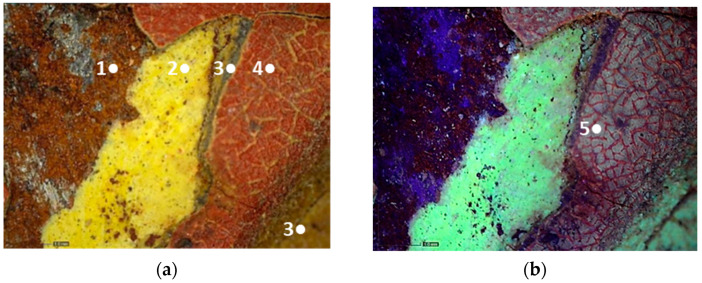
Optical microscope image of the layering on the carriage: (**a**) Image collected in VIS light, showing the substrate (1), the bright yellow layer (2), the yellow-brown layer (3), that is above or under the red layer, and the red layer (4); (**b**) Image collected in UV light, where the finishing layer-with a bluish fluorescence can be observed (5) above the colored layers.

The analysis that was carried out with optical microscopy made it possible to observe the complex stratigraphy of the carriage’s paints, which was mainly characterized by the superimposition of four paint layers (Figure 8).

Starting from the bottom, the stratigraphy can be described as follows:

4. The support: it is mostly metal-based, while some areas are made of wood.
5. The bright yellow layer: it is present in a few areas of the carriage, such as the back panel of the box letter, the rear axles of wheels, and the metal axes under the left panel of the box letter.
6. The yellow-brown layer.
7. The red layer.
8. The finishing layer.

**Figure 9:**
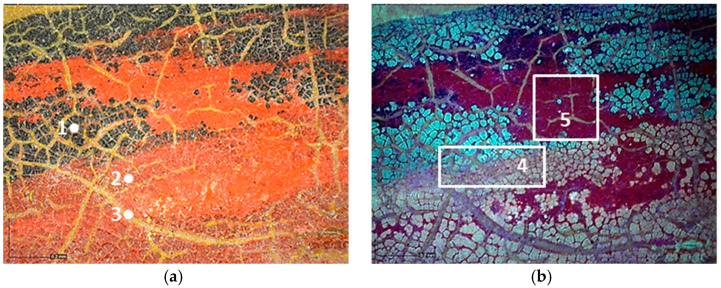
Optical microscope image (200× magnification) of the area corresponding to the postal service’s symbol. (**a**) Image collected in VIS light showing the cracking process with thinner (1–2) and thicker cracks (3) that reach the yellow paint; (**b**) Image collected in UV light highlighting the islands of different sizes and shapes where the finishing layer is present (4), and an area with different fluorescence in which the layer is absent (5).

The finishing layer is characterized by a bluish-yellow fluorescence. It shows widespread cracking and degradation signs which seem to be due to anomalous contractions of the material itself (Figure 9). 

**Figure 10:**
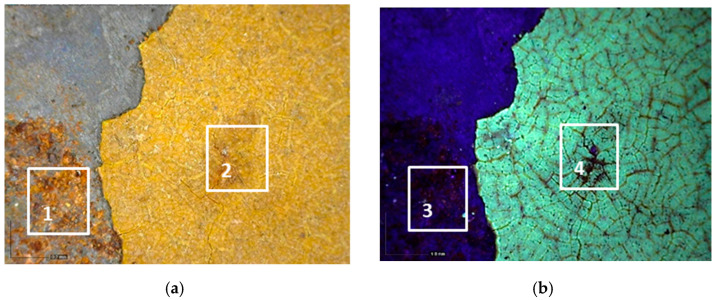
Optical microscope images (200× magnification) of one of the metal panels: corrosion of the laminate (1–3), causing the darkening of the yellow paint (2–4). (**a**) Image collected in VIS light; (**b**) Image collected in UV light.

The craquelure was presumably induced by the imperfect adhesion between the finishing layer and the underlying ones but also by exposure to UV radiation and a high level of relative humidity [34]. In the overlapping areas of the red paint on the yellow one, such cracks are not limited to the surface only, but reach the underlying yellow layer. All types of branched cracking are according to variable, straight, or concentric crosslinks. These crosslinks intersect each other, forming islands of various sizes and shapes. Some areas of the carriage, such as the panels, show that the symbols are characterized by the absence of the finishing layer (Figure 10). Indeed, in these areas, it is possible to detect the underlying paints, which have a different fluorescence when observed in ultraviolet light (Figure 10b).

Moreover, the metal support of the carriage appears to be corroded, and some areas of the superimposed paint layers appear darker, presumably due to corrosion products of the underlying laminate (Figure 10).

**Figure 11:**
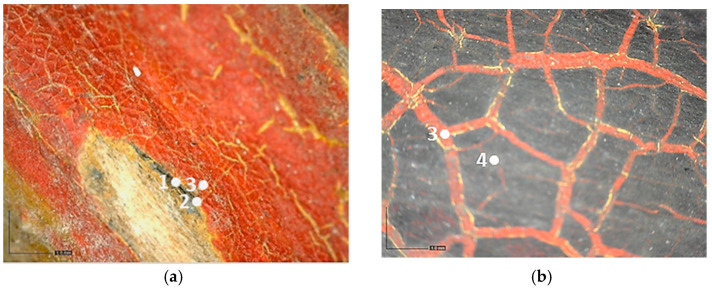
Optical microscope images of the areas showing the black paint. (**a**) Image (50× magnification) collected in VIS light showing the black paint (1) under the yellowish brown (2) and red (3) layers; (**b**) Image (50× magnification) collected in VIS light showing the black layer (4) above the red one (3).

Black paint was used exclusively for decorations (Figure 11).

### 2.3. Fourier Transform Infrared Coupled Attenuated Total Reflectance (FT-IR ATR)

**Figure 12:**
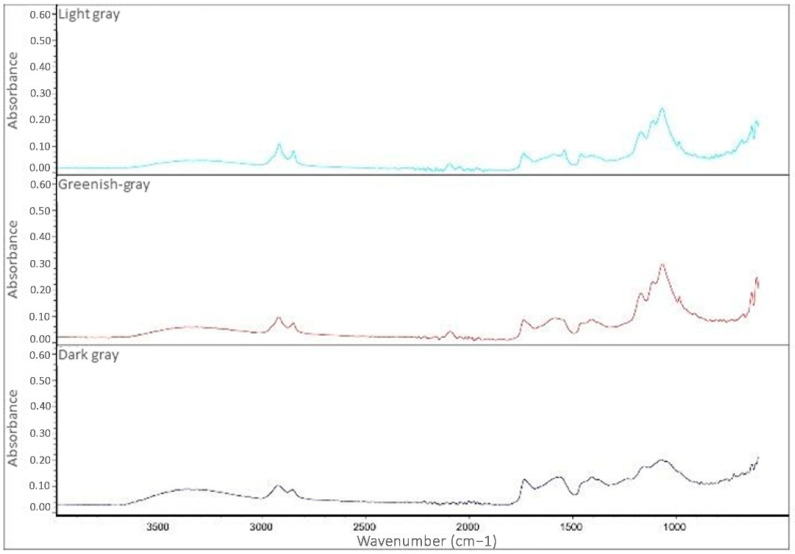
FT-IR ATR spectra of the three grey paints of the cart.

**Figure 13:**
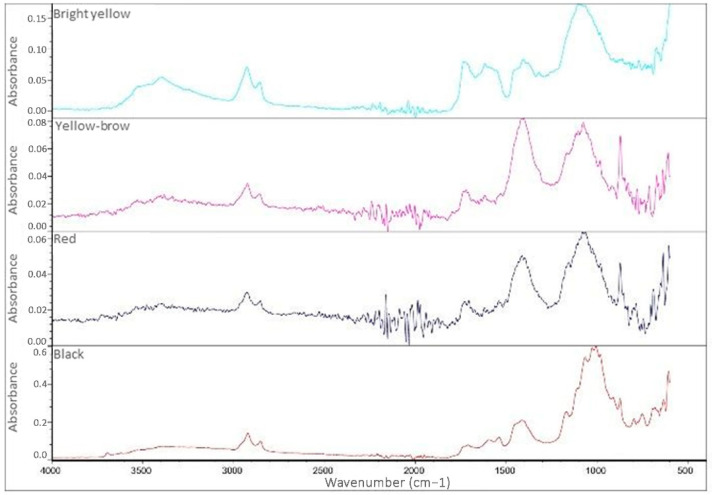
FT-IR ATR spectra of the colored paints of the carriage.

The colored paints of both the carriage and the wagon are characterized by similar FT-IR ATR spectra (Figure 12 and Figure 13).

The main difference is in the bands at 1400 and 870 cm^−1^, which are almost completely absent in the black paint of the carriage and the paints of the wagon. These bands can be linked to the presence of carbonates, which are therefore found in greater amounts in the yellow and red paints of the carriage compared to its total or almost absent in the grey-black paints. The interpretation of spectra allowed for the assignment of the following bands [35,36,37,38,39,40,41,42,43]:

o 3500–3400 cm^−1^: stretching of the O-H bonds, present in linseed oil and also in some silicates;
o 2950–2800 cm^−1^: stretching of the C-H bonds present in the aliphatic chains of the oil;
o 1730 cm^−1^: stretching of the C=O bond of the esters present in the siccative oil;
o 1600–1400 cm^−1^: stretching of the COO- group of carboxylates, the presence of which is due to the degradation of the drying oil in the presence of metal cations;
o 1400 and 870 cm^−1^: asymmetric stretching of the carbonate group (CO_3_^2−^);
o 1163 cm^−1^: asymmetric stretching of the C-C-O bonds of the esters present in the oils;
o 1080–1020 cm^−1^: vibrational stretching of the Si-O and Si-O-Si bonds, which is typical of silicates.

The paints of the two vehicles are, therefore, characterized by the presence of aged drying oils, silicates, and sometimes carbonates, while the bands related to carboxylates (1600–1400 cm^−1^) are due to the aging process and degradation of the drying oil. 

**Figure 14:**
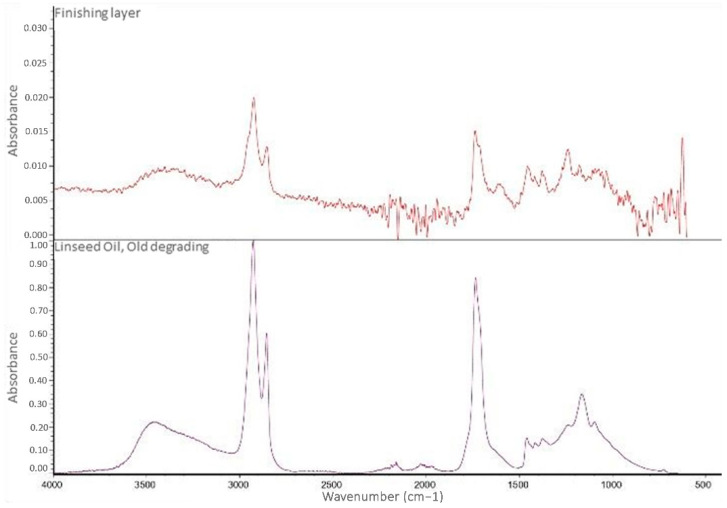
FT-IR ATR spectrum of the finishing layer extracted using MEK (**above**) and the spectrum of degraded linseed oil (**bottom**).

The analysis of the finishing layer present on the surface of the carriage required the extraction of the finishing layer from the sample with methyl ethyl ketone (MEK). The spectrum acquired on the extracted materials showed the characteristic bands of an oil-based component and carboxylates, similar to the spectra collected on the colored paints (Figure 14).

### 2.4. Scanning Electron Microscopy Coupled with Energy Dispersive Spectroscopy (SEM-EDS)

#### 2.4.1. The Cart

SEM/EDS data of the cart’s paints allows important differences between the light grey paint to be highlighted; hence, the most external and recent one compared with the innermost ones, with the dark grey paint directly applied on the wood.

The dark grey paint is characterized by the presence of zinc, barium, and carbon (Table 3). Arsenic is present in low percentages and cannot be related to the color of the paint. For this reason, it can be linked to the wooden support possibly having undergone a biocide treatment. Indeed, arsenic is a poisonous compound that is used for the disinfestation of insects, while copper is generally used for bacteria. The high percentages of carbon suggest that it could have been used with a pigment of organic origin (e.g., carbon black).

The SEM-EDS analysis carried out on the outermost paint layers (Table 4) made it necessary to exclude carbon from the elements’ count in order to obtain more significant information on the elements present in smaller quantities, such as silicon, potassium, and calcium. It is, however, likely that the pigment used to give the grey color can be identified as carbon black, as in the previous paint’s analysis. 

The elemental analysis carried out on the intermediate paint layer (Table 4; Spectrum 3), i.e., the greenish-grey one, allowed a greater presence of zinc and barium to be noticed, which were probably used to make the dark grey paint lighter. The EDS analysis collected on the light grey layer (Table 4; Spectra 1–2) was characterized by the presence of zinc and titanium. The presence of titanium is of the utmost significance since it is absent in all other grey paints. This element can be linked to the use of titanium white together with zinc white to obtain the desired color gradation and opacity of the paint. Additionally, the presence of iron can be related to the chromatic alterations resulting from the solubilization and leaching of the iron corrosion products of the wagon’s structure.

In addition, the paints related to the iron axes (sample 18) showed a yellowish-brown color that was characterized by the presence of titanium and a high iron content (Table 5). Eventually, titanium was related to the use of titanium white, as already stated in the results obtained on the light-grey paint, while the iron was related to the corrosion of the metal axes containing superimposed paint layers. 

#### 2.4.2. The Carriage

The results of the SEM-EDS analysis allowed for the identification of the pigments based on the evaluation of detected chemical elements.

The bright yellow paint on the top of the metal support is characterized by a high amount of lead, which is not present in the other examined paints (Table 6). This paint represents the original layer of the carriage, whose yellow color is attributable to the presence of lead compounds.

SEM-EDS analysis on the yellow-brown paint (second layer) allowed for the presence of silicates to be noticed. Due to the extensive corrosion of the metal panels (Figure 10), iron was excluded from the elemental analysis of the yellow paint, as it was attributable to both corrosion products and the use of iron-based pigments (Table 7). However, the lack of other chromophores indicates that the pigment of the paint is likely yellow earth. 

The SEM images of the red paint (Table 8) show the significant cracking forming regular blocks (third paint layer). It is a characteristic feature of the cracking induced by an imperfect adhesion between the red layer and the yellow paint layer underneath [34,44]. In the elemental analysis, carbon was omitted because its high value did not allow the semiquantitative determination of other elements, such as zinc, barium, sulfur, and calcium.

The results reported in Table 8, together with the elemental analysis acquired on a detail of the surface layer (Table 9), underline the presence of an organic compound. This information allows for the hypothesis that the paint was characterized by an organic dye that provided the red color. Moreover, higher magnification enabled the observation of uniform particles without a specific structure that could be attributed to materials not relevant to the paint. Indeed, the observation in the backscattered electrons (BSE) mode showed the presence of light particles on a dark matrix that was relevant to organic compounds. The presence of these inorganic particles was found in all paints and is attributable to earthy dust or plaster on the walls of the storage environment.

The black paint used for decorations on the red one is characterized by purely organic components, as shown by the SEM/EDS analysis (Table 10). No chromophore elements were detected. Thus, the black color was most likely given by the carbon present in the pigments of organic origin (e.g., carbon black). Traces of silicates, zinc, barium, sulfur, and calcium were also found, similar to the other paints.

## 3. Discussion

The data provided by the multi-analytical investigation on the two vehicles, together with information found in the literature, have made it possible to obtain useful information regarding the composition of the matrices of paints, thus enabling their dating.

Multispectral imaging and optical portable microscopy allowed for the detection of the cart’s stratigraphy, which appeared to have a simpler structure compared to the carriage. Indeed, it consists of three layers: the dark grey paint in direct contact with the wooden support, as well as the original paint; the greenish-grey paint, namely the intermediate one; the light grey paint, namely the outermost one. On the other hand, the carriage shows a stratigraphy consisting of the metal support, four paint layers (a yellow one, a yellow-brown one, a red one, and a black one corresponding to decorations only), and a finishing layer that is visible under UV radiation.

The chromophore element, which gives the grey color to the three paints, has been identified as carbon due to the use of a dye or an organic pigment.

SEM-EDS analysis allowed for the detection of high concentrations of lead in the innermost yellow paint layer, which could be related to the use of a yellow lead pigment. As previously mentioned, this pigment was used until the 1940s in Europe [4,6,7,8,9,14,15]. Zinc has also been found in the greenish-grey intermediate layer. It could be linked to the use of zinc white (zinc oxide) and/or lithopone, which have been used mainly since the late 19th century until the first half of the 20th century [5,6,7,8,14,16,18]. The presence of barium could be related to the use of lithopone (a mixture of barium sulfate and zinc sulfide) but also to the use of natural barite: a white pigment used as a filler. Further fillers that were detected in the vehicle’s layer matrices consist of silicates and carbonates [4,6,7,14,18]. Furthermore, the chromophore element provided the grey color of the three paints, which were identified as carbon, while the presence of arsenic could be related to an insect biocide for the wooden support [30]. The outermost paint (the light-grey one) is mainly characterized by the presence of titanium, related to the use of titanium white. This pigment was discovered in the 1920s and was increasingly used from the 1940s [4,6,7,8,9,14,15]. The elemental analysis allowed for the identification of zinc in high concentrations in the outermost paint layer, suggesting the use of a composite titanium pigment made up of titanium and zinc whites. This information allowed for the dating of the paint presumably after the 1920s, when titanium white was discovered, and before the 1970s, when titanium oxide was no longer used as a composite pigment [16]. Eventually, SEM-EDS analysis also detected iron, especially in the outermost paint layers. The presence of this element is due to the corrosion of the metal axes of the cart structure, leading to the formation of chromatic alterations, which was also observed through multispectral imaging. The FT-IR ATR analysis highlighted the presence of silicates and carbonates while also detecting the characteristic bands of degraded oils in both the finishing layer, and the paint layer underneath. In all the paints, the presence of an oily binder could suggest that they might date before the 1940s when paints based on synthetic resins took over [4,6,7,8,9,14,15]. Whereas FT-IR spectroscopy detected the vibration bands associated with chemical bonds and oils of similar functional groups, it was not possible to define the exact type of oil. The most commonly used oily binder has been linseed oil, and it may have been used for the production of studied paints [4,6,7,8,9,14,15]. Despite these considerations, all oily binders were used up until the first half of the 20th century, when they were then completely replaced by synthetic resins [4,6,7,8,9,14,15].

The FT-IR ATR carried out on the carriage showed the presence of silicates and carbonates, as well as the characteristic bands of oil and carboxylates, highlighting once again the presence of degradation products of oily substances [43]. As in the cart, the presence of peaks at 1600–1400 cm^−1^ is related to the stretching of the COO- a group of carboxylates, which highlights the significant degradation of the drying oil. 

While FT-IR ATR spectroscopy did not allow the exact identification of the pigments or dyes used for the red and black paints in the carriage, the SEM-EDS analysis allowed for the detection of a high concentration of lead in the innermost yellow paint layer, which could be related to the use of a yellow lead pigment. As previously mentioned, this pigment was used until the 1940s in Europe [14].

The bright yellow paint was lost in most areas of the carriage and was replaced by yellow-brown paint. Even though the SEM-EDS analysis of this paint’s samples required the exclusion of iron since the high values could be related either to the corrosion of the metal sheets or the use of yellow ochre, this is one of the most used yellow pigments in history [45].

The two other paintings of the carriage, i.e., the red paint and the black one were used for decorations. They are characterized by a high carbon content, suggesting that both paints were related to the use of dyes or organic pigments.

The SEM-EDS analysis also highlighted the presence of calcium, silicon, barium, sulfur, and zinc in all the paint layers. The presence of calcium and silicon can be attributed, respectively, to the use of carbonates and silicates, while barium and sulfur enabled the hypothesis of barite. All these compounds were used in the paints’ formulation as fillers to improve stability and decrease production costs. Zinc is attributable to the use of zinc oxide: a pigment that was discovered in the late 18th century and became common in the late 19th century. However, the presence of zinc and barite did not allow the exclusion of the use of lithopone: another opaque white pigment that has been used since 1880 [5,6,7,8,14,16,18].

In conclusion, the diagnostic investigations allowed the identification of the carriage’s paints, which are oil-based paints containing pigments based on zinc, barium, and lead for the bright yellow paint only. The characterization suggested the dating of the paints before 1950 when the oil paints were replaced by paints based on synthetic resins, which were discovered in the first decades of the 20th century. The absence of titanium allows for estimating the dating of the paints before the 1940s.

## 4. Materials and Methods

### 4.1. The Vehicles

As previously mentioned, this article is focused on the study of protective paints used on two vehicles. They are conserved at the Museum für Kommunikation in Frankfurt and were used by the German postal service at the turn of the two centuries. 

The first one is a cart for telecommunications services and is dated approximately 1920–1940 (Figure 1). This cart was constructed in the Reichspost era and was continuously used by the postal service throughout the German Democratic Republic (GDR). The cart was found in the garden of a former postal employee in 2015. It was partially sunk into the ground, where it presumably stood for decades after having been taken out of service.

The second vehicle is a carriage of the German postal service, dated around 1880 (Figure 2). The vehicle was found in 1999 when the King’s family house was demolished. It is currently stored at the Museum für Kommunikation in Frankfurt. It is supposed to have been used for the postal service between Schramberg and Rottwell until 1910.

### 4.2. Portable Optical Microscopy

A portable optical microscope Dino-Lite AM411-FVW (Dino-Lite Europe, Almere, The Netherlands) was used to investigate the surface morphology. It was performed using different magnifications, from 40× to 220× (where X = enlargement), and three illumination modes: visible (VIS), ultraviolet (UV), and ranking (VIS-RAD) light. 

### 4.3. Multispectral Imaging

Multispectral imaging was carried out through VIS, UV, and infrared (IR) spectrum bands. Fluorescence UV was used to identify and characterize the presence of film-forming substances on the surface, while mid- and near-infrared spectrum bands verified the presence of different materials based on their interaction with infrared radiation. The investigation was carried out with the Madatec multispectral system (Madatec, Pessano con Bornago, Italy) consisting of a Samsung NX500 28.2 MP BSI CMOS camera. Ultraviolet fluorescence was observed using CR230B-HP Madatec UV spotlights (365 nm), HOYA UV-IR filter cut 52, and Yellow 495 52 mm F-PRO MRC 022. Infrared reflectography images were obtained using an 850 nm filter. 

### 4.4. FT-IR ATR Spectroscopy

Spectroscopy FT-IR was performed to characterize the paint materials at the functional-group level. The spectra were gained using the Nicolet Summit FT-IR spectrometer (Thermo Fisher Scientific, Waltham, MA, USA) and equipped with the Everest™ Diamond ATR accessory, with an instrumental resolution of 8 cm^−1^; 32 scans were performed on each sample, and the respective spectra were analyzed using the database library and the scientific literature. The analysis was carried out on 18 samples taken from the areas highlighted in Table 11. Samples (1 × 1 mm) were taken from the selected areas using a scalpel to sample the whole layer structure of the stratigraphy.

### 4.5. SEM-EDS

The SEM-EDS technique was performed to carry out morphological, structural, and chemical analysis on the same samples used for FT-IR ATR analysis. The investigations were carried out with a VEGA3-Tescam instrument (Tescan, Brno, Czech Republic) coupled with an Inca 300 Energy Dispersive X-ray (Oxford Instruments Analytical, High Wycombe, United Kingdom) microanalysis system. Samples were analyzed in a high vacuum with a beam potential of 30 KeV: a suitable intensity to be able to perform the elemental composition in EDS acquisition. The SEM observations were performed in secondary electrons (SE) together with backscattered electrons (BSE). Furthermore, operating a high vacuum allowed for reliable quantitative values and even light elements.

## 5. Conclusions

The exposure of objects to the environment led to changes in their properties, functionality, and integrity. For this reason, protective paints have been traditionally used to protect surfaces from the external environment. They have undergone great evolutions over the years. Specifically, the transformations increased at the turn of the 19th and 20th centuries, thanks to the discovery and development of new compounds, together with the withdrawal from the market of toxic elements. 

Oily binders were used until the first half of the 20th century, while synthetic binders were discovered in the 1920s, completely replacing oily binders in the second half of the century. Between the 19th and 20th centuries, significant developments occurred in the pigments’ manufacturing process as well. These changes were mainly represented by the progressive disposal of lead-based pigments and the discovery of new pigments, such as zinc white, lithopone, and titanium white.

Due to the potential of such binders and pigments to be used as markers for the dating of objects and artworks, the present study aimed at characterizing the paints of the cart and the carriage belonging to the Museum für Kommunikation in Frankfurt while estimating their dating and identifying the authentic materials to be preserved. The paints of the two vehicles might have been applied before 1950, as they are oil-based. The paints of the carriage and the innermost paints of the cart might be dated before 1940, before the widespread use of titanium white. In addition, the outermost paints of the cart are characterized by the presence of titanium and are thus dated between the 1920s and the 1950s.

In conclusion, the analytical investigations allowed the carriage and the cart to be dated, confirming their authenticity and also gaining significant information on the paints used in Germany for the decoration of vehicles in the postal service. Furthermore, obtaining information on the compositional nature and historical importance of the paints is essential for directing any restoration project which aims to preserve the historical and testimonial importance of these vehicles.

## Figures and Tables

**Table 1:** Summary of images obtained in different areas of the cart with multispectral analysis using visible light (VIS), ultraviolet fluorescence, and infrared reflectography (IR).

Area	VIS	UV FLUORESCENCE	IR 850
Left panel, top left corner	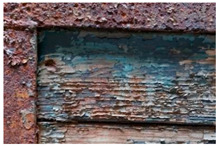	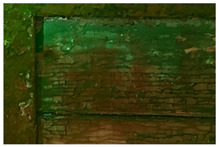	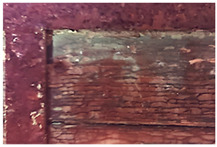
Right panel, area where the plate was posted	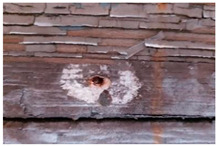	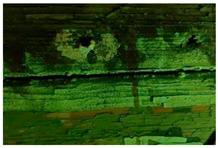	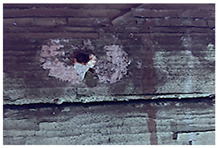
Rear door, top left corner	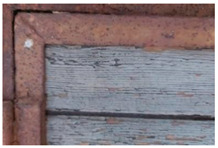	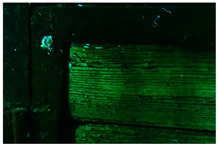	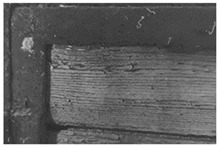
Right panel, plate	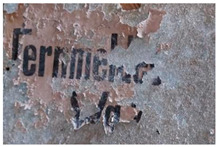	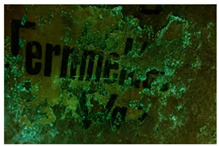	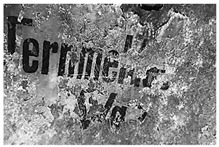

**Table 2:** Multispectral images showing different areas of the carriage: visible light (VIS), ultraviolet fluorescence (UV), and infrared reflectography (IR).

Area	VIS	UV FLUORESCENCE	IR 850
Symbol on the right side	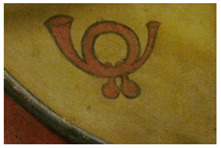	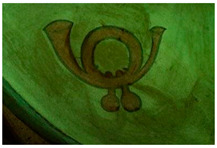	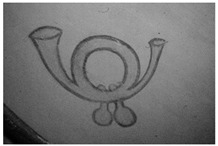
Symbol on the left side	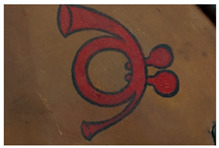	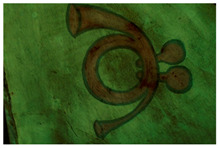	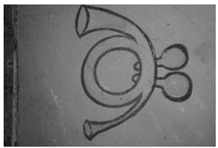
Rear panel	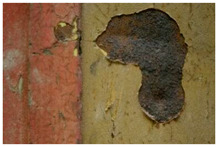	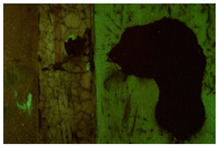	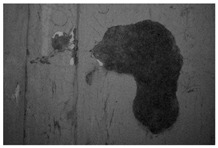
Left-side panel	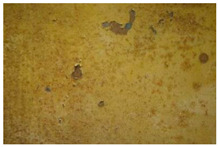	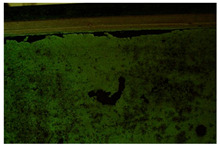	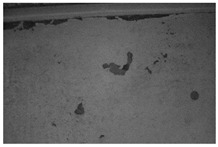
Rear-left wheel	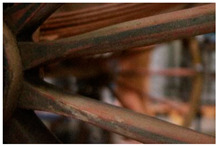	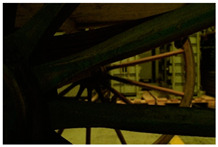	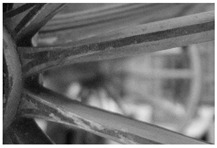
Left-side flap	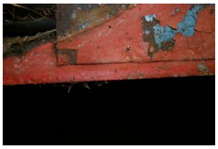	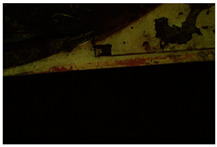	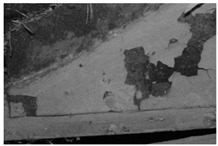
Rear wheel axle	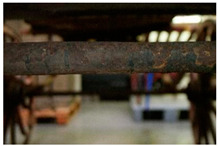	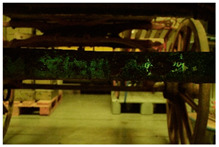	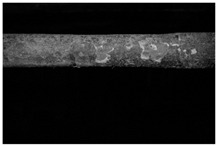

**Table 3:** SEM image collected on the grey inner paint of the cart. In the table, the data obtained by the EDS analysis are reported.

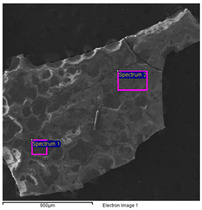	**Spectrum**	**C**	**O**	**Mg**	**Si**	**S**	**Ca**	**Fe**	**Zn**	**As**	**Ba**
	Spectrum 1	45.7	36.2	0.2	0.3	4.8	0.2	0.8	4.2	0.3	7.3
	Spectrum 2	44.8	32.6	0.4	0.3	5.4	0.3	1.1	5.5	0.4	9.3

Quantitative values in wt% (±0.2).

**Table 4:** SEM/EDS analysis of sample 16. Data obtained by the EDS analyses. The analyzed sample is characterized by the two outermost layers: the light gray layer (1) on which spectra 1 and 2 were collected; the intermediate layer (2) on which spectrum 3 was collected.

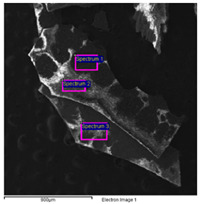	**Spectrum**	**O**	**Al**	**Si**	**S**	**K**	**Ca**	**Ti**	**Fe**	**Zn**	**Ba**
	Spectrum 1						1.4	29.7	13.0	55.9	
	Spectrum 2	66.3	1.8	3.9	2.7	0.3	1.1	13.6	3.4	6.9	

Qualitative values in wt% (±0.2).

**Table 5:** SEM image collected on sample 18. In the table, the EDS analysis is reported. Spectra 1–3 were collected on the brownish paint; Spectrum 2 was acquired on the underlying grey layer.

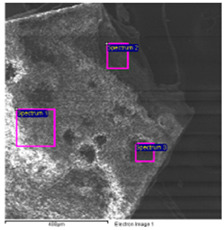	**Spectrum**	**C**	**O**	**Al**	**Si**	P	**S**	**Cl**	**K**	**Ca**	**Ti**	**Fe**	**Zn**	**Ba**
	Spectrum 1	-	65.5	2.9	2.3	-	2.9	-	0.3	1.2	5.3	17.9	1.7	-
	Spectrum 2	38.0	31.3	0.5	-	-	4.7	-	-	0.4	-	1.3	13.7	10.1
	Spectrum 3	-	63.1	2.6	2.3	0.4	3.8	0.3	0.3	1.6	6.8	16.6	2.3	-

Quantitative values in wt% (±0.2).

**Table 6:** SEM image collected on the bright yellow paint of the carriage at high magnification. In the table, the data obtained from the EDS analysis are reported.

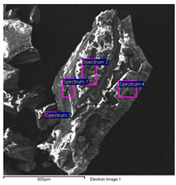	**Spectrum**	**O**	**Al**	**Si**	**S**	**K**	**Ca**	**Fe**	**Zn**	**As**	**Br**	**Ba**	**Pb**
	Spectrum 1	62.6	1.4	-	-	-	1.4	-	13.0	-	-	-	20.8
	Spectrum 2	62.2	1.5	1.9	4.1	0.5	1.6	9.7	11.3	0.0	-	4.0	3.2
	Spectrum 3	44.8	-	-	-	-	2.4		13.4	-	0.0	5.4	34.0
	Spectrum 4	74.7	2.6	2.5	3.5	-	1.6	1.8	6.0	-	-	2.0	5.3

Quantitative values in wt% (±0.2).

**Table 7:** SEM image collected on the yellow-brown paint of the carriage. In the table the reported data were obtained from the EDS analysis (excluding iron).

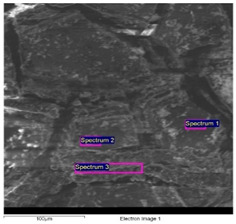	**Spectrum**	**C**	**O**	**Al**	**Si**	**S**	**K**	**Ca**	**Zn**	**Ba**
	Spectrum 1	52.4	46.3	0.3	0.2	0.2	-	0.5	0.3	-
	Spectrum 2	-	77.0	-	1.3	9.4	0.6	10	1.7	-
	Spectrum 3	24.8	51.5	-	3.8	4.8	-	9.2	1.3	4.6

Quantitative values in wt% (±0.2).

**Table 8:** SEM image of the red paint of the carriage. In the table are reported the data obtained from the EDS analysis.

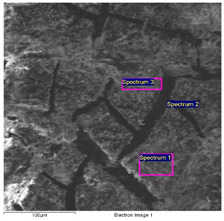	**Spectrum**	**O**	**Mg**	**Al**	**Si**	**S**	**K**	**Ca**	**Fe**	**Zn**	**Ba**
	Spectrum 1	78.0	-	2.5	4.0	5.3	0.5	4.3	1.2	4.2	-
	Spectrum 2	70.6	0.6	1.3	2.0	11.2	-	11.2	0.8	2.3	-
	Spectrum 3	52.0	-	2.5	5.2	11.0	0.4	3.4	2.8	6.2	16.5

Quantitative values in wt% (±0.2).

**Table 9:** SEM image collected on the red paint of the carriage at high magnification. In the table, the data obtained from the EDS analysis are reported.

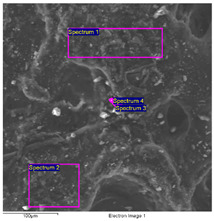	**Spectrum**	**C**	**O**	**Al**	**Si**	**S**	**Ca**	**Fe**	**Zn**	**Ba**
	Spectrum 1	47.6	44.0	-	-	0.4	7.0	0.4	-	0.6
	Spectrum 2	-	78.3	1.5	1.8	-	18.4	-	-	-
	Spectrum 3	43.4	31.0	-	-	3.8	0.8	0.9	1.0	19.0
	Spectrum 4	35.0	29.0	-	-	6.3	0.7	0.8	1.0	27.0

Quantitative values in wt% (±0.2).

**Table 10:** SEM image collected on sample 3. In the table, the data obtained from the EDS analysis of the black paint are reported.

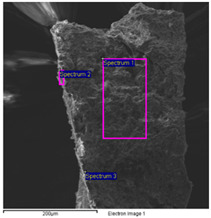	**Spectrum**	**C**	**O**	**Al**	**Si**	**S**	**Ca**	**Fe**	**Zn**	**Ba**
	Spectrum 1	39.0	51.4	0.7	1.2	1.1	4.4	0.2	0.5	1.6

Quantitative values in wt% (±0.2).

**Table 11:** Sampling areas.

Vehicle	Vehicle’s Side	Sampling Areas
Carriage	Right side and rear axle (sample 6)	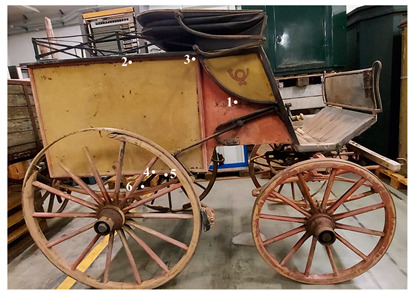
Carriage	Rear panel of the box letter	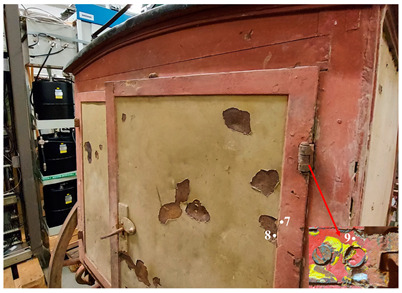
Carriage	Left side, flap under the seat	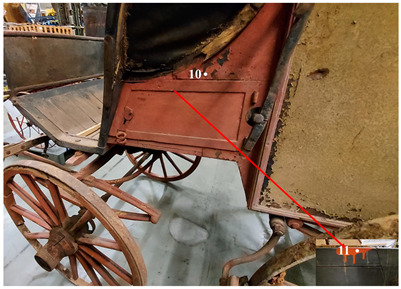
Carriage	Front panel	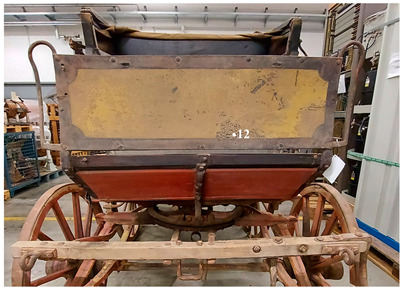
Carriage	Left symbol (was detached; found inside the left flap)	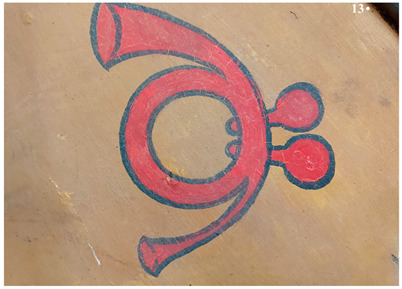
Cart	Left panel	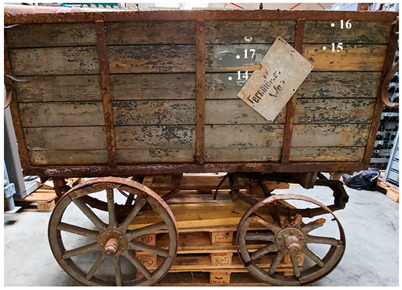
Cart	Frontal panel	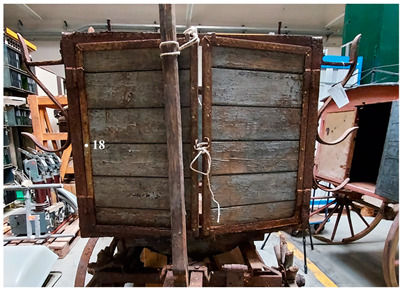

## Data Availability

Publicly available datasets were analysed in this study to compare the acquired FT-IR spectra with already available spectra of standard materials. Data can be found here: https://spectra.chem.ut.ee/(accessed on 5 November 2022).
